# Vitamin D Every Day to Keep the Infection Away?

**DOI:** 10.3390/nu7064170

**Published:** 2015-05-29

**Authors:** Juliana de Castro Kroner, Andrea Sommer, Mario Fabri

**Affiliations:** 1 Department of Dermatology, University of Cologne, 50937 Cologne, Germany; E-Mails: jdcastro@smail.uni-koeln.de (J.C.K.); andrea.sommer@uk-koeln.de (A.S.); 2 Center for Molecular Medicine Cologne, University of Cologne, 50937 Cologne, Germany

**Keywords:** Vitamin D, macrophage, dendritic cell, T cell, infection and immunity

## Abstract

Within the last decade, vitamin D has emerged as a central regulator of host defense against infections. In this regard, vitamin D triggers effective antimicrobial pathways against bacterial, fungal and viral pathogens in cells of the human innate immune system. However, vitamin D also mediates potent tolerogenic effects: it is generally believed that vitamin D attenuates inflammation and acquired immunity, and thus potentially limits collateral tissue damage. Nevertheless, several studies indicate that vitamin D promotes aspects of acquired host defense. Clinically, vitamin D deficiency has been associated with an increased risk for various infectious diseases in epidemiological studies; yet, robust data from controlled trials investigating the use of vitamin D as a preventive or therapeutic agent are missing. In this review, we summarize the current knowledge regarding the effect of vitamin D on innate and acquired host defense, and speculate on the difficulties to translate the available molecular medicine data into practical therapeutic or preventive recommendations.

## 1. Introduction

**Figure 1:**
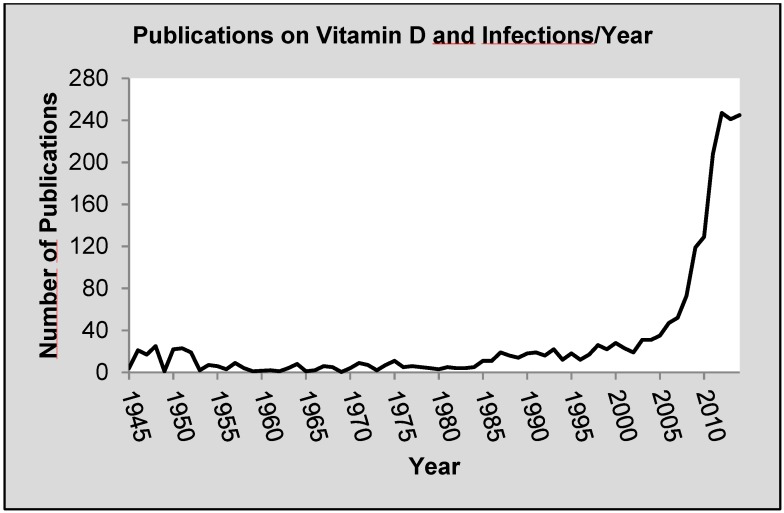
Number of scientific publications addressing “vitamin D” and “infection(s)” per year (until 2014). Data from PubMed (US National Library of Medicine) search engines [14].

The pivotal role of vitamin D in the regulation of calcium homeostasis and bone health has been known since long ago. However, it was later discovered that the vitamin D receptor (VDR) is functional in tissues that are not involved in calcium metabolism [1]. In fact, microarray analyses indicate that vitamin D regulates, directly or indirectly, up to 5% of the human genome and induces physiologic responses in ≥36 different cell types [1,2]. A renewed interest in novel vitamin D functions resulted in an enormous number of publications addressing these extra-skeletal effects of vitamin D in the last decade, including the effect on the human immune system and infectious diseases (Figure 1). Hossein-nezhad and colleagues recently showed that vitamin D supplementation *in vivo* regulated the expression of 291 genes in white blood cells, known to interfere with more than 160 distinct biological pathways. Among these genes, those associated with immunological responses had a prominent position, supporting the idea of vitamin D as an important immune regulator [3].

Despite labeled as a “vitamin”, in fact, vitamin D is a secosteroid hormone. Thus, beside the possibility of nutritional intake from cod liver oil, fatty fishes (e.g., salmon and tuna), eggs and vitamin D-fortified products, the main source of vitamin D is synthesis in the skin from 7-dihydroxycholesterol upon UVB irradiation. In this instance, the pandemic occurrence of vitamin D deficiency, which affects approximately one billion people in the world, is considered a consequence of our predominantly urbanized indoor lifestyle [4]. Both sun-induced and dietary vitamin D are hydroxylated firstly to 25-hydroxy-vitamin D (25D), mainly in the liver, by cytochrome P450 enzymes as the CYP27a1- and CYP2r1-hydroxylases [5]. Then, 25D is modified by the 25-hydroxyvitamin D-1-α-hydroxylase (CYP27B1), mainly in the kidney, to generate bioactive 1,25-dihydroxyvitamin D (1,25D). Both 25D and 1,25D are transported in the blood linked to the vitamin D binding protein (DBP). Because the affinity of 1,25D to the VDR is 1000-fold higher when compared to 25D, 1,25D is considered the main activator of VDR-mediated effects [6]. Importantly, vitamin D is not only converted from 25D into 1,25D in the kidney, but is also locally activated by the CYP27B1-hydroxylase in many different tissues, including brain, smooth muscle, breast and prostate, as well as cells of the immune system. Thus, vitamin D can act not only in an endocrine, but also in a paracrine, intracrine or autocrine manner [7,8]. In this process, the DBP seems to critically regulate the bioavailability of 25D for monocytes, DCs and T cells [9,10,11,12].

The facts that (i) immune cell functions are critically regulated by bioactive 1,25D; and (ii) immune cells metabolically participate in the generation of 1,25D from serum 25D, clearly document the importance of vitamin D in shaping immune responses. Meanwhile, observational studies reported that vitamin D deficiency is associated with an increased risk for various infectious diseases, including tuberculosis, HIV, respiratory tract and HCV infections [8], thereby fuelling discussions as to whether vitamin D deficiency is causally linked to an increased risk for infectious diseases. However, data from controlled clinical trials remain poor and show contradictory results [13]. In this review, we discuss the current knowledge of vitamin D immune regulatory functions in the context of infectious diseases, highlighting its specific implications to innate and acquired host defense. Moreover, we speculate on the difficulties and limitations to translate the current molecular medicine knowledge into practical therapeutic recommendations.

## 2. Vitamin D in Innate Host Defense

**Figure 2:**
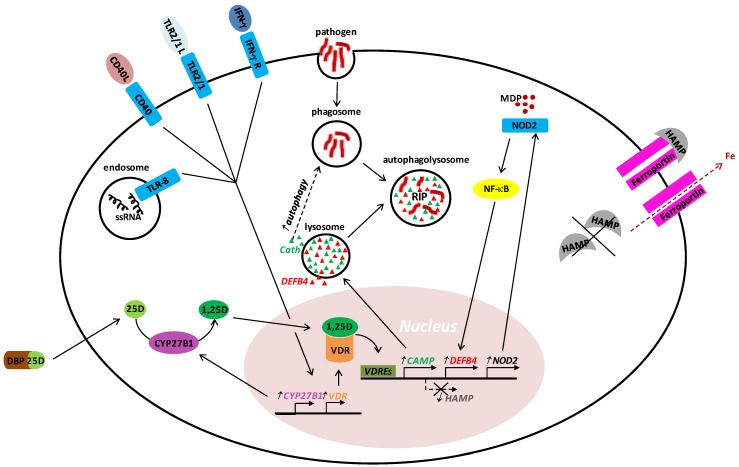
Proposed model of the vitamin D-dependent antimicrobial pathway. Toll-like receptor (TLR)-2/1 ligand, TLR-8 ligand (innate immune mechanisms), interferon-gamma (IFN-γ) and CD40 ligand (CD40L) (acquired immune mechanisms) induce antimicrobial response in human monocytes/macrophages through different signaling pathways; yet they converge on the upregulation of 25-hydroxyvitamin D-1-α-hydroxylase (CYP27B1) and vitamin D receptor (VDR). CYP27B1 hydroxylates 25-hydroxyvitamin D (25D) into the bioactive 1,25-dihydroxyvitamin D (1,25D), triggering VDR mediated upregulation of antimicrobial peptides (cathelicidin and human β-defensin 2 (DEFB4)), as well as nucleotide-binding oligomerization domain-containing protein 2 (NOD2), which indirectly contributes to further DEFB4 increase. At the same time, the 1,25D-bound VDR mediates downregulation of hepcidin (HAMP). HAMP favors the cellular export of iron and makes the intracellular compartment inconvenient for the survival/proliferation of pathogens. In addition, cathelicidin promotes autophagy, which enhances autophagolysosomal fusion and antimicrobial activity.

As members of the innate immune system, monocytes, macrophages and dendritic cells (DCs) provide a crucial line of defense against infectious agents. Here, the central role of these cells relies on two main aspects: first, they display special germ-line encoded pattern recognition receptors (PRRs), e.g., toll-like receptors (TLRs), that are able to recognize conserved microbial motifs and initiate cellular programs for pathogen killing and induction of inflammation; second, they use internalized material for antigen presentation to T cells, providing an important interface with cells of the acquired immune system. With respect to antimicrobial innate responses, we have contributed to the understanding of the role of vitamin D in human host defense by characterizing an autocrine vitamin D pathway in human monocytes/macrophages after stimulation by TLR2/1 ligand, T-cell-derived interferon-gamma (IFN-γ) or T-cell-expressed CD40 ligand [15,16,17,18] (Figure 2). These receptors initiated a signaling cascade that induced the upregulation of VDR and CYP27B1, resulting in conversion of 25D to 1,25D. Subsequently, 1,25D triggered VDR activation and its dependent downstream induction of antimicrobial peptides cathelicidin and human β-defensin 2 (DEFB4), autophagy and phagosome maturation, resulting in intracellular killing of pathogens, as shown for *Mycobacterium tuberculosis* [15,16,17,18,19], *M. marinum* [20] *and M. leprosy* [21]. In addition to direct activation of the vitamin D host response, T cell cytokines differentially influenced TLR2/1-mediated induction of this pathway: while the Th1-cytokine IFN-γ cooperated with a TLR2/1 ligand in inducing CYP27B1 activity, the Th2-cytokine IL-4 promoted the upregulation of CYP24A1, which catalyzes vitamin D to an inactive form [22]. As a result, IFN-γ enhanced, while IL-4 suppressed the vitamin D-mediated induction of cathelicidin, DEFB4 and autophagy. In addition, DEFB4 can be indirectly enhanced by vitamin D upregulation of the nucleotide-binding oligomerization domain-containing protein 2 (NOD2): the activation of this PRR by muramyl dipeptide from Gram-negative/-positive bacteria induces the NF-κB-mediated transcription of DEFB4 in humans [23,24]. Interestingly, cathelicidin and DEFB4 are direct targets of the VDR in humans, yet not in rodents. The human promoter of cathelicidin contains three vitamin D response elements (VDRE), while the mouse has none [25]. DEFB4, which contains one VDRE in its promoter, has no homolog in mouse [16].

The vitamin D innate host response is also active in many other cells, as is the case of keratinocytes, gastrointestinal/bronchial epithelial cells, decidual and trophoblastic cells [8,26]. Accordingly, vitamin D bactericidal action is broad-reaching and can provide protection against various pathogens, especially in airway infections. Yin and colleagues, for example, have reported enhanced cathelicidin-mediated killing of *Pseudomonas aeruginosa* and *Bordetella bronchiseptica* in bronchial epithelial cells upon vitamin D treatment [27]. Yet, of note, the mechanisms mediating vitamin D-dependent antimicrobial responses are not the same for all cell types. In keratinocytes, IL-17 has been reported to enhance 1,25D-dependent induction of cathelicidin via Act-1/MEK-ERK activation, even though IL-17 alone had no influence on cathelicidin levels [28]. In contrast, Kao *et al.* identified IL-17 as a potent cytokine directly inducing DEFB4 expression in the airway epithelium, a process reportedly mediated by JAK and NF-κB signaling [29]. Intriguingly, however, during IL-17 co-stimulation of keratinocytes, 1,25D restricted the expression of DEFB4 and other IL-17 target genes, like IL-6 and IL-8 [28]. In 2007, Schauber *et al.* reported that in keratinocytes of wounded skin, TLR2/6 ligands and TGF-β can trigger expression of VDR responsive genes involved in the induction of cathelicidin and other antimicrobial peptides; among such genes, TLR2 and the TLR co-receptor CD14 [30]. This could explain the ability of keratinocytes to rapidly sense and respond to TLR2 ligands, providing fast protection in wounds against bacterial and fungal infections [30]. Remarkably, cathelicidin also displays antiviral effects, thereby supporting host response against influenza virus or HIV [8]. In this instance, Campbell and Spector observed inhibition of HIV replication in macrophages via vitamin D induction of autophagy and phagosomal maturation, processes reportedly dependent on cathelicidin expression triggered by TLR8 ligands [31,32] (Figure 2). Thus, it is tempting to speculate that if butyrate treatment (histone deacetylase inhibitors) enhances 1,25D induction of cathelicidin, as previously demonstrated in human keratinocytes [33], drugs targeting histone acetylation might constitute an interesting strategy to boost vitamin D beneficial antimicrobial effects in the context of different infections. Even though the mouse homolog of cathelicidin, CRAMP, is not under direct control of 1,25D, Muehleisen *et al.* reported that it was induced by parathyroid hormone (PTH), and 1,25D induced the PTH receptor 1 in mouse keratinocytes [34]. In their studies, mice that were on dietary vitamin D restriction responded with an elevation in PTH, yet showed an increased risk for group A *Streptococcus* infection if they lacked 1,25D. Thus, the authors speculated that PTH signaling via its type 1 receptor compensate for inadequate vitamin D during activation of antimicrobial peptide production [34]. In contrast to these results, Love *et al.* found that the vitamin D induction of cathelicidin in human defense cells enhanced group A *Streptococcus* resistance to killing via LL-37-mediated induction of streptococcal virulence factors [35].

Recently, it was shown that cathelicidin and DEFB4 are not the only antimicrobial peptides regulated by vitamin D, but also hepcidin [36] (Figure 2). This protein is known to modulate the tissue distribution of iron by suppressing the ferroportin (cell membrane protein)-mediated export of cellular iron. Bacchetta *et al.* reported that both 25D and 1,25D downregulate the expression of hepcidin in cultured hepatocytes and monocytes [36]. Such downregulatory effect may constitute an interesting tool against intracellular pathogens as *M. tuberculosis*, *Salmonella typhimurium*, *Leishmania donovani*, *Chlamydia psittaci*, *C. trachomatis*, and *Legionella pneumophila* [37]: reduced expression of hepcidin leads to increased export of iron and reduced concentrations inside the cellular environment. Since bacterial survival and growth is dependent of iron, this may restrict proliferation of intracellular pathogens; on the other hand, extracellular iron would be oppositely regulated, which could have an undesired effect in the control of systemic infections [38].

Besides antimicrobial activity, the production of pro-inflammatory cytokines by macrophages and DCs is important to orchestrate immune responses. The overproduction of these cytokines, on the other hand, leads to unresolved inflammation and tissue injury. Zhang *et al.* reported that Vitamin D upregulates MKP-1 to mediate suppression of pro-inflammatory cytokines in monocytes/macrophages [39]. However, most studies describing suppressive effects refer to time/dose-dependent events that are attributed to vitamin D feedback mechanisms to reduce tissue damage - among them, 1,25D mediated downregulation of PRR (as TLR2, TLR4, Dectin-1 and mannose receptor) [40,41] and upregulation of CYP24-hydroxylase [42,43]. Furthermore, it remains controversial whether vitamin D really suppresses or even enhances pro-inflammatory cytokines in the context of infections. For instance, vitamin D increased secretion of IL-1β in macrophages through direct transcriptional mechanisms both in *M. tuberculosis*-infected and non-infected macrophages [44]. Nevertheless, IL-1β secretion was only observed in infected cells, because IL-1β maturation was ultimately dependent on *M. tuberculosis* induction of NLRP3 inflammasome-mediated caspase-1 activation [44]. Interestingly, macrophage secretion of IL-1β induced DEFB4 production by co-cultured epithelial cells, which in turn, promoted antimicrobial activity in the surrounding macrophages. Moreover, vitamin D was reported to boost the expression of other key host defense cytokines and chemokines, including IL-6, IL-23, IL-12, IL-8, TNF-α, CCL3, CCL4, CCL8, CCL20 and MIP1α in human macrophages [44,45]. Of note, increased secretion of TNF-α and IL-6 by vitamin D-treated DCs has been observed [46,47,48], yet not consistently interpreted as such [49]. Thus, vitamin D seems to trigger antimicrobial host defense and enhance early inflammatory reactions necessary to cell recruitment and efficient coordination of immune responses, meanwhile preventing extensive inflammation and tissue destruction by negative feedback mechanisms.

## 3. Vitamin D in Acquired Host Defense 

Vitamin D modulates acquired host responses by directly acting on T cells [9,50,51] and indirectly by regulating DC functions [52]. DCs express VDR, as well as CYP27a1 and CYP27b1 [53,54,55,56], thereby locally generating relevant amounts of bioactive 1,25D. Most *in vitro* studies used 1,25D and revealed that the phenotype of 1,25D-treated monocyte-derived DCs is characterized by low expression of antigen-presenting and co-stimulatory molecules, as well as sustained CD14 expression [47,57,58,59,60]. This DC phenotype is relatively resistant to maturation and shows a reduced ability to trigger allogenic T cell proliferation [57,59,60,61]. Moreover, 1,25D treatment promotes DC secretion of IL-10, a regulatory T cell (Treg) promoting cytokine, over IL-12 family members, which are Th1/Th17-driving cytokines [46,47,48,52,57,58,59,62].

In contrast to DCs, human resting memory and naïve CD4^+^ and CD8^+^ T cells reportedly express VDR only at low levels. Nevertheless, VDR expression is upregulated upon T cell activation, which correlates with the level of T cell stimulation, and is stabilized against proteasomal degradation by 1,25D [63,64,65,66,67,68,69,70]. Simultaneously, T cells upregulate CYP27b1 [51,67]. Thus, T cells are able to generate 1,25D from 25D to modulate T cell effector functions in an autocrine fashion, independently of paracrine 1,25D secretion by antigen-presenting cells (APCs) [51,67,68,71]. Both CD4^+^ and CD8^+^ memory T cell proliferation, irrespective as to whether antigen-, leptin- or IL-2-induced, is inhibited by 1,25D. This has been linked to a block in transition from early to late GI phase and lowering of IL-2 production through NF-AT/AP-1 complex [6,68,69,72,73,74]. Furthermore, *in vitro* assays suggest that 1,25D, by directly acting on T cells, restricts Th1/Th17 cell differentiation and favors Th2 differentiation [9,70,75,76,77,78,79,80,81]. Moreover, 25D and 1,25D generated CTLA4^+^ and FoxP3^+^, IL-10-secreting Tregs [9,10,80,82]. Taken together, in addition to DC-mediated effects, vitamin D also favors anti-inflammatory T cells phenotypes via its direct effect on T cells.

The observed immunosuppressive effects of vitamin D on DCs and T cells *in vitro* are in line with clinical findings that vitamin D deficiency is linked with the risk for autoimmune diseases [83]. However, vitamin D deficiency has also been associated with a higher incidence of infections [83], in which similar DC- and T cell-mediated mechanisms are important to mount an effective host response. A possible explanation is that vitamin D promotes innate host defense, while balancing the acquired host defense to limit excessive inflammation. Nevertheless, vitamin D was shown to be required for induction of phospholipase C-γ1 (PLC-γ), which is important for full T cell activation during initiation of human naïve T cell response to antigens [65]. Remarkably, it takes *ca.* 48 h between antigen recognition and full upregulation of PLC-γ [65]. In this regard, it has been speculated that, if the innate immune system is able to clear the infection rapidly, the vitamin D-mediated delay of full T cell activation puts a break on excessive cell proliferation to avoid immunopathology [26,65]. Both 25D and 1,25D influences lymphocyte trafficking by upregulation of CCR10 expression *in vitro* on naïve and effector T cells, thereby contributing to T cell migration towards the skin [51,67]. Additionally, Baeke *et al.* detected reduced surface expression of lymphoid organ-homing receptors CCR7, and correspondingly upregulated inflammatory homing receptor CCR5 on T cells treated with a 1,25D analog [84].

Most studies used classical allogenic T cell responses (mixed lymphocyte reactions), the *in vitro* models of allograft rejection, to evaluate effect of vitamin D on APC-T cell interactions. It remains possible that the induction of a T cell response to a foreign-antigen during infection is divergently regulated, because it differently depends on antigen binding, antigen uptake, intracellular processing (including degradation by proteasome and lysosomal-associated proteases), loading on MHC molecules and also on the quality of MHC:peptide complexes [85]. For example, Olliver *et al*. demonstrated that vitamin D enhanced expression of CD80, CD86 and MHC class II and migratory marker CCR7 on DCs upon maturation with live pneumococci [84]. Nevertheless, vitamin D, as reported by others, also skewed the DC-mediated T helper response from an inflammatory Th1/Th17 phenotype towards a Treg cell phenotype in their study. A hypothetical mechanism by which vitamin D could have an enhancing effect on presentation of pathogen-associated antigens is by inducing autophagy [86]. Autophagy is a highly conserved cellular pathway degrading intracellular engulfed material through phagolysosomal fusion. Interestingly, autophagy enhances the MHC class I- and MHC class II-mediated antigen presentation in cases of viral and bacterial [87,88,89] antigens. Vitamin D is a known important inducer of autophagy, yet whether this is linked to antigen presentation is speculative.

## 4. Mouse Models of Infection

In line with the described *in vitro* observations, vitamin D receptor (VDR) knockout (KO) mice are more susceptible to Th1-mediated autoimmunity, for instance, to multiple sclerosis and inflammatory bowel disease. In addition, 1,25D treatment contributes to prolonged allograft survival in mouse models [90,91,92]. In contrast, VDR KO mice have decreased production of Th2, making them more resistant to Th2-cell driven experimental asthma [93]. In light of the *in vitro* and *in vivo* observations in models of autoimmunity, transplantation and chronic inflammatory disease, one could hypothesize that vitamin D impairs the ability of the host response to infection in mouse models [92]. In particular, the host response against intracellular infections, in which CD4^+^ Th1 immune responses are fundamental to activate macrophages and to further instruct CD8^+^ cytotoxic T cells. However, *in vitro* investigations of different mouse models, including VDR KO mice, 1,25D-treated wild type (WT) mice, vitamin D deficient or sufficient WT mice, reveal conflicting results. VDR KO mice infected with the intracellular pathogen *L. major* indeed showed smaller lesions and lower parasite burden, which was related to decreased nitric oxide and an increased arginase-1 expression [94,95]. However, the CD4^+^ and CD8^+^ Th1 responses were not altered in these mice. On the contrary, VDR KO mice compared to controls showed only a slight delay in clearance of Listeria infection in young and old mice, even though old VDR KO mice had lower memory CD4^+^ and CD8^+^ T cell numbers [96]. Moreover, VDR KO mice displayed a relatively unchanged ability compared to WT mice to clear *Mycobacterium tuberculosis*, *Candida albicans* and *Herpes simplex* 1 [90,91,97]. In contrast, Chlamydia infection, which is also controlled by a Th1-mediated immune response, was more prolonged in VDR KO mice, because of a reduced expression of the anti-inflammatory leucocyte elastase inhibitor [98].

Studies on extracellular pathogens show likewise controversial results. Chen *et al.* found that VDR KO mice had lower *Citrobacter rodentium* load and faster pathogen clearance linked to a higher number of IL-22-producing innate lymphoid cells (ILCs), which positively influenced antibacterial peptide expression in epithelial cells [99]. Furthermore, vitamin D decreased effector and memory CD8^+^ T cell differentiation and trafficking potential at early stages of T cell activation against *choriomeningitis virus* [100]. In contrast, host resistance of VDR KO mice compared to WT was unchanged when infected with *Shistosoma mansoni* or *Bordetella pertussis* [101]. Furthermore, Nguyen *et al.* demonstrated that nutritional vitamin D deficiency leads to an increased Th2 host response to *Aspergillus fumigatus* in mice via direct regulation of OX40L [102]. Of interest, in *A. fumigatus* infected patients, this observation correlated with attenuated TGF-β^+^ Treg numbers and was associated with a better disease outcome. The aforementioned study by Muehleisen *et al.* linked vitamin D deficiency to an increased risk of infection by group A *Streptococci* [34]. In summary, it seems that the effect of vitamin D on the host response and the outcome of an infection is critically dependent on the infecting agent.

While a positive effect of vitamin D on innate host defense seems clear, the effect on acquired host defense is not fully elucidated. Some mouse studies have unraveled mechanisms by which vitamin D promotes acquired host responses. Enioutina *et al.* report in vaccination protocols that co-administration of 1,25D in mice did not influence antigen presentation capabilities [103,104]. However, 1,25D promoted trafficking of antigen-loaded myeloid DCs from draining lymph node to other secondary lymphoid organs, thereby supporting the activation of a larger pool of T cells. Only transiently vitamin D-exposed DCs properly activate naïve T cell responses, but not bone marrow-derived DCs under constant vitamin D exposure. Strikingly, Lim *et al.* recently reported in a mouse model of systemic candida infection that divergent 1,25D levels could be one explanation for the contradictory results in *in vitro* and *in vivo* studies. They showed that low doses of 1,25D induced protective immunity against systemic candida infection, mediated by IFN-γ, TNF-α, IL-17. In contrast, higher 1,25D doses led to lower survival rates of candida-infected mice compared to controls. These data correlated with former *in vitro* observations using PBMCs [41] and suggest that high doses of 1,25D are counterproductive in infectious conditions. This could also explain, why treatment with high doses of 1,25D had no positive effect for mice suffering from systemic candidiasis [101,105]. Taken together, we conclude that several studies identify immune boosting effects of vitamin D and shed some light on possible explanations for differing effects of vitamin D on infections compared to autoimmune and graft-*versus*-host disease.

## 5. Comments on Therapeutic Intervention

The link between low 25D serum levels and the increased incidence of various infectious diseases, in combination with the identification of molecular mechanisms *in vivo* and *in vitro*, by which vitamin D regulates immune responses, have encouraged attempts to use vitamin D as a preventive agent and adjuvant therapeutic for infectious diseases (reviewed in detail in [13]). However, the conclusions drawn from clinical studies addressing the use of vitamin D on prevention and treatment of infectious diseases, including tuberculosis, respiratory tract, HCV and HIV infections, have been inconsistent [13]. The reasons are diverse [106]. One of the central issues of the study designs is the uncertainty regarding doses and dosing intervals. This is directly related to the fact that clearly defined target 25D serum levels, which could even differ in distinct infectious diseases, are missing. In addition, the increase of 25D serum levels by a given dose of vitamin D is significantly correlated with the pretreatment baseline 25D levels [107]. Moreover, the target 25D level might be also patient specific, given that genetic variations in the DBP and the VDR critically influence the end-organ bioavailability and activation of vitamin D at various levels [9,11,108,109]. In this regard, it is also possible that serum 25D not bound to the DBP, that is, albumin-bound and free 25D, plays a greater role than generally believed [106]. Furthermore, potential differences between vitamin D2 *vs*. vitamin D3 treatment remain to be clarified [13,110]. Of note, some studies have been complicated by the facts that the study population was not 25D deficient at baseline [13], or that the placebo group showed a similar increase in 25D serum levels as the serum-treated group. Vitamin D, in contrast to many other drugs investigated in clinical trials, is easily obtainable *over-the-counter* and/or from the sun. Another aspect that might compromise the outcome of clinical trials is that pathogens can corrupt vitamin D host defense pathways [21], which might also explain some of the differences observed in mouse studies investigating different pathogens [101].

The use of vitamin D becomes even more complicated in the context of a general population-wide vitamin D supplementation for prevention of infectious, or other extra-skeletal diseases. Given that vitamin D levels are determined by *common* and *individual* factors, different groups have varying recommendations of intake. In the absence of vitamin D supplementation, regular diet does not contribute much to the vitamin D status in most populations, except in uncommon dietary habits. In contrast, evolutionally, the human skin may have developed a system that fine-tunes vitamin D synthesis, allowing us to obtain overall optimal levels at a given latitude and an outdoor life style. However, critical common factors that determine the degree of vitamin D production in the skin include the variations in sunlight exposure associated with the degree of air pollution, latitude, and season [111,112]. As a consequence, UVB radiation is not sufficient to ensure adequate year-round *de novo* vitamin D synthesis in most parts of the world [113]. In addition, UVB radiation is also the main cause of human skin cancer, thus it is difficult, if not impossible, to make a general recommendation to expose the skin to the sun for sufficient vitamin D synthesis. Furthermore, the degree of cutaneous vitamin D production is dependent on individual factors. For example, it is known that highly-pigmented and elderly individuals are at higher risk to vitamin D deficiency for they are less able to photosynthesize it: dark skin people need six-times more UVB exposure to reach the same vitamin D serum levels of Caucasians [114] and individuals over 70-year-old synthesize more than two-fold less the amount of vitamin D they did before their 20s [115]. The same applies to the obese population, recent reports show 57% lower cutaneous production than the normal weight population [116]. In addition, obese individuals present reduced vitamin D intestinal absorption [116]. Other conditions that interfere with the gut absorbance of lipids, as in inflammatory bowel disease, constitute additional factors compromising the contribution from diet to our daily vitamin D quota [117]. The influence of all these different aspects on the vitamin D status, in combination with patient- and disease-specific target 25D serum levels, raises concerns as to whether a general recommendation of vitamin D supplementation or treatment is possible at all without a complex risk stratification taking individual genetic, biometric and environmental aspects into account. In summary, despite the progress made in identifying molecular mechanisms by which vitamin D regulates host defense in mice and humans, the lack of robust data from rigorously designed clinical trials make transfer of studies from bench to bedside very difficult [13,83,118]. It remains to be seen if the identification of biomarkers, for instance with the help of large scale microarrays and ChIP sequencing [119], will help to group individuals corresponding to their responsiveness to vitamin D [120,121], and if this will finally translate into robust data from clinical trials and into personalized medicine approaches.

## References

[1] Norman A.W. (2008). From vitamin D to hormone D: Fundamentals of the vitamin D endocrine system essential for good health. Am. J. Clin. Nutr..

[2] Hossein-nezhad A., Holick M.F. (2013). Vitamin D for health: A global perspective. Mayo Clin. Proc..

[3] Hossein-nezhad A., Spira A., Holick M.F. (2013). Influence of vitamin D status and vitamin D3 supplementation on genome wide expression of white blood cells: A randomized double-blind clinical trial. PLoS ONE.

[4] Zhang R., Naughton D.P. (2010). Vitamin D in health and disease: Current perspectives. Nutr. J..

[5] Bikle D.D. (2010). Vitamin D: Newly discovered actions require reconsideration of physiologic requirements. Trends Endocrinol. Metab..

[6] Tsoukas C.D., Provvedini D.M., Manolagas S.C. (1984). 1,25-dihydroxyvitamin d 3: A novel immunoregulatory hormone. Science.

[7] Hewison M. (2010). Vitamin D and the intracrinology of innate immunity. Mol. Cell Endocrinol..

[8] Lang P.O., Samaras N., Samaras D., Aspinall R. (2013). How important is vitamin D in preventing infections?. Osteoporos. Int..

[9] Jeffery L.E., Wood A.M., Qureshi O.S., Hou T.Z., Gardner D., Briggs Z., Kaur S., Raza K., Sansom D.M. (2012). Availability of 25-hydroxyvitamin D3 to apcs controls the balance between regulatory and inflammatory t cell responses. J. Immunol..

[10] Jeffery L.E., Burke F., Mura M., Zheng Y., Qureshi O.S., Hewison M., Walker L.S., Lammas D.A., Raza K., Sansom D.M. (2009). 1,25-dihydroxyvitamin D3 and il-2 combine to inhibit T cell production of inflammatory cytokines and promote development of regulatory T cells expressing ctla-4 and foxp3. J. Immunol..

[11] Chun R.F., Lauridsen A.L., Suon L., Zella L.A., Pike J.W., Modlin R.L., Martineau A.R., Wilkinson R.J., Adams J., Hewison M. (2010). Vitamin D-binding protein directs monocyte responses to 25-hydroxy- and 1,25-dihydroxyvitamin D. J. Clin. Endocrinol. Metab..

[12] Grzybowski A., Pietrzak K. (2012). Tadeusz reichstein (1897‒1996): A cofounder of modern steroid treatment in dermatology. Clin. Dermatol..

[13] Kearns M.D., Alvarez J.A., Seidel N., Tangpricha V. (2015). Impact of vitamin D on infectious disease. Am. J. Med. Sci..

[14] PubMed. http://www.ncbi.nlm.nih.gov/pubmed/.

[15] Liu P.T., Stenger S., Li H., Wenzel L., Tan B.H., Krutzik S.R., Ochoa M.T., Schauber J., Wu K., Meinken C. (2006). Toll-like receptor triggering of a vitamin D-mediated human antimicrobial response. Science.

[16] Liu P.T., Schenk M., Walker V.P., Dempsey P.W., Kanchanapoomi M., Wheelwright M., Vazirnia A., Zhang X., Steinmeyer A., Zugel U. (2009). Convergence of il-1beta and vdr activation pathways in human tlr2/1-induced antimicrobial responses. PLoS ONE.

[17] Fabri M., Stenger S., Shin D.M., Yuk J.M., Liu P.T., Realegeno S., Lee H.M., Krutzik S.R., Schenk M., Sieling P.A. (2011). Vitamin D is required for ifn-gamma-mediated antimicrobial activity of human macrophages. Sci. Transl. Med..

[18] Klug-Micu G.M., Stenger S., Sommer A., Liu P.T., Krutzik S.R., Modlin R.L., Fabri M. (2013). Cd40l and ifn-gamma induce an antimicrobial response against M. Tuberculosis in human monocytes. Immunology.

[19] Yuk J.M., Shin D.M., Lee H.M., Yang C.S., Jin H.S., Kim K.K., Lee Z.W., Lee S.H., Kim J.M., Jo E.K. (2009). Vitamin D3 induces autophagy in human monocytes/macrophages via cathelicidin. Cell Host. Microbe.

[20] Sato E., Imafuku S., Ishii K., Itoh R., Chou B., Soejima T., Nakayama J., Hiromatsu K. (2013). Vitamin D-dependent cathelicidin inhibits mycobacterium marinum infection in human monocytic cells. J. Dermatol. Sci..

[21] Liu P.T., Wheelwright M., Teles R., Komisopoulou E., Edfeldt K., Ferguson B., Mehta M.D., Vazirnia A., Rea T.H., Sarno E.N. (2012). Microrna-21 targets the vitamin D-dependent antimicrobial pathway in leprosy. Nat. Med..

[22] Edfeldt K., Liu P.T., Chun R., Fabri M., Schenk M., Wheelwright M., Keegan C., Krutzik S.R., Adams J.S., Hewison M. (2010). T-cell cytokines differentially control human monocyte antimicrobial responses by regulating vitamin D metabolism. Proc. Natl. Acad. Sci. USA.

[23] Reich K.M., Fedorak R.N., Madsen K., Kroeker K.I. (2014). Vitamin D improves inflammatory bowel disease outcomes: Basic science and clinical review. World J. Gastroenterol..

[24] Wang T.T., Dabbas B., Laperriere D., Bitton A.J., Soualhine H., Tavera-Mendoza L.E., Dionne S., Servant M.J., Bitton A., Seidman E.G. (2010). Direct and indirect induction by 1,25-dihydroxyvitamin D3 of the nod2/card15-defensin beta2 innate immune pathway defective in crohn disease. J. Biol. Chem..

[25] Gombart A.F., Borregaard N., Koeffler H.P. (2005). Human cathelicidin antimicrobial peptide (camp) gene is a direct target of the vitamin D receptor and is strongly up-regulated in myeloid cells by 1,25-dihydroxyvitamin d3. FASEB J..

[26] White J.H. (2012). Vitamin D metabolism and signaling in the immune system. Rev. Endocr. Metab. Disord..

[27] Yim S., Dhawan P., Ragunath C., Christakos S., Diamond G. (2007). Induction of cathelicidin in normal and cf bronchial epithelial cells by 1,25-dihydroxyvitamin D(3). J. Cyst. Fibros..

[28] Peric M., Koglin S., Kim S.M., Morizane S., Besch R., Prinz J.C., Ruzicka T., Gallo R.L., Schauber J. (2008). Il-17a enhances vitamin D3-induced expression of cathelicidin antimicrobial peptide in human keratinocytes. J. Immunol..

[29] Kao C.Y., Chen Y., Thai P., Wachi S., Huang F., Kim C., Harper R.W., Wu R. (2004). Il-17 markedly up-regulates beta-defensin-2 expression in human airway epithelium via jak and nf-kappab signaling pathways. J. Immunol..

[30] Schauber J., Dorschner R.A., Coda A.B., Buchau A.S., Liu P.T., Kiken D., Helfrich Y.R., Kang S., Elalieh H.Z., Steinmeyer A. (2007). Injury enhances tlr2 function and antimicrobial peptide expression through a vitamin D-dependent mechanism. J. Clin. Invest..

[31] Campbell G.R., Spector S.A. (2012). Vitamin D inhibits human immunodeficiency virus type 1 and mycobacterium tuberculosis infection in macrophages through the induction of autophagy. PLoS Pathog..

[32] Campbell G.R., Spector S.A. (2012). Toll-like receptor 8 ligands activate a vitamin D mediated autophagic response that inhibits human immunodeficiency virus type 1. PLoS Pathog..

[33] Schauber J., Oda Y., Buchau A.S., Yun Q.C., Steinmeyer A., Zugel U., Bikle D.D., Gallo R.L. (2008). Histone acetylation in keratinocytes enables control of the expression of cathelicidin and cd14 by 1,25-dihydroxyvitamin d3. J. Invest. Dermatol..

[34] Muehleisen B., Bikle D.D., Aguilera C., Burton D.W., Sen G.L., Deftos L.J., Gallo R.L. (2012). Pth/pthrp and vitamin D control antimicrobial peptide expression and susceptibility to bacterial skin infection. Sci. Transl. Med..

[35] Love J.F., Tran-Winkler H.J., Wessels M.R. (2012). Vitamin D and the human antimicrobial peptide ll-37 enhance group a streptococcus resistance to killing by human cells. MBio.

[36] Bacchetta J., Zaritsky J.J., Sea J.L., Chun R.F., Lisse T.S., Zavala K., Nayak A., Wesseling-Perry K., Westerman M., Hollis B.W. (2014). Suppression of iron-regulatory hepcidin by vitamin D. J. Am. Soc. Nephrol..

[37] Cherayil B.J. (2011). The role of iron in the immune response to bacterial infection. Immunol. Res..

[38] Chun R.F., Liu P.T., Modlin R.L., Adams J.S., Hewison M. (2014). Impact of vitamin D on immune function: Lessons learned from genome-wide analysis. Front. Physiol..

[39] Zhang Y., Leung D.Y., Richers B.N., Liu Y., Remigio L.K., Riches D.W., Goleva E. (2012). Vitamin D inhibits monocyte/macrophage proinflammatory cytokine production by targeting mapk phosphatase-1. J. Immunol..

[40] Khoo A.L., Chai L.Y., Koenen H.J., Oosting M., Steinmeyer A., Zuegel U., Joosten I., Netea M.G., van der Ven A.J. (2011). Vitamin D(3) down-regulates proinflammatory cytokine response to mycobacterium tuberculosis through pattern recognition receptors while inducing protective cathelicidin production. Cytokine.

[41] Khoo A.L., Chai L.Y., Koenen H.J., Kullberg B.J., Joosten I., van der Ven A.J., Netea M.G. (2011). 1,25-dihydroxyvitamin D3 modulates cytokine production induced by candida albicans: Impact of seasonal variation of immune responses. J. Infect. Dis..

[42] Hewison M. (2010). Vitamin D and the immune system: New perspectives on an old theme. Endocrinol. Metab. Clin. North Am..

[43] Avila E., Diaz L., Halhali A., Larrea F. (2004). Regulation of 25-hydroxyvitamin D3 1alpha-hydroxylase, 1,25-dihydroxyvitamin D3 24-hydroxylase and vitamin d receptor gene expression by 8-bromo cyclic amp in cultured human syncytiotrophoblast cells. J. Steroid. Biochem. Mol. Biol..

[44] Verway M., Bouttier M., Wang T.T., Carrier M., Calderon M., An B.S., Devemy E., McIntosh F., Divangahi M., Behr M.A. (2013). Vitamin D induces interleukin-1beta expression: Paracrine macrophage epithelial signaling controls m. Tuberculosis infection. PLoS Pathog..

[45] Larcombe L., Orr P., Turner-Brannen E., Slivinski C.R., Nickerson P.W., Mookherjee N. (2012). Effect of vitamin D supplementation on mycobacterium tuberculosis-induced innate immune responses in a canadian dene first nations cohort. PLoS ONE.

[46] Kleijwegt F.S., Laban S., Duinkerken G., Joosten A.M., Zaldumbide A., Nikolic T., Roep B.O. (2010). Critical role for tnf in the induction of human antigen-specific regulatory T cells by tolerogenic dendritic cells. J. Immunol..

[47] Unger W.W., Laban S., Kleijwegt F.S., van der Slik A.R., Roep B.O. (2009). Induction of treg by monocyte-derived dc modulated by vitamin D3 or dexamethasone: Differential role for pd-l1. Eur. J. Immunol..

[48] Chamorro S., Garcia-Vallejo J.J., Unger W.W., Fernandes R.J., Bruijns S.C., Laban S., Roep B.O., t Hart B.A., van Kooyk Y. (2009). Tlr triggering on tolerogenic dendritic cells results in tlr2 up-regulation and a reduced proinflammatory immune program. J. Immunol..

[49] Wobke T.K., Sorg B.L., Steinhilber D. (2014). Vitamin D in inflammatory diseases. Front. Physiol..

[50] Mahon B.D., Gordon S.A., Cruz J., Cosman F., Cantorna M.T. (2003). Cytokine profile in patients with multiple sclerosis following vitamin D supplementation. J. Neuroimmunol..

[51] Sigmundsdottir H., Pan J., Debes G.F., Alt C., Habtezion A., Soler D., Butcher E.C. (2007). Dcs metabolize sunlight-induced vitamin D3 to “program” T cell attraction to the epidermal chemokine ccl27. Nat. Immunol..

[52] Penna G., Amuchastegui S., Giarratana N., Daniel K.C., Vulcano M., Sozzani S., Adorini L. (2007). 1,25-dihydroxyvitamin D3 selectively modulates tolerogenic properties in myeloid but not plasmacytoid dendritic cells. J. Immunol..

[53] Brennan A., Katz D.R., Nunn J.D., Barker S., Hewison M., Fraher L.J., O'Riordan J.L. (1987). Dendritic cells from human tissues express receptors for the immunoregulatory vitamin D3 metabolite, dihydroxycholecalciferol. Immunology.

[54] Hewison M., Freeman L., Hughes S.V., Evans K.N., Bland R., Eliopoulos A.G., Kilby M.D., Moss P.A., Chakraverty R. (2003). Differential regulation of vitamin D receptor and its ligand in human monocyte-derived dendritic cells. J. Immunol..

[55] Fritsche J., Mondal K., Ehrnsperger A., Andreesen R., Kreutz M. (2003). Regulation of 25-hydroxyvitamin D3-1 alpha-hydroxylase and production of 1 alpha,25-dihydroxyvitamin D3 by human dendritic cells. Blood.

[56] Kundu R., Chain B.M., Coussens A.K., Khoo B., Noursadeghi M. (2014). Regulation of cyp27b1 and cyp24a1 hydroxylases limits cell-autonomous activation of vitamin D in dendritic cells. Eur. J. Immunol..

[57] Penna G., Adorini L. (2000). 1 alpha,25-dihydroxyvitamin D3 inhibits differentiation, maturation, activation, and survival of dendritic cells leading to impaired alloreactive t cell activation. J. Immunol..

[58] Piemonti L., Monti P., Sironi M., Fraticelli P., Leone B.E., Dal C.E., Allavena P., Di C.V. (2000). Vitamin D3 affects differentiation, maturation, and function of human monocyte-derived dendritic cells. J. Immunol..

[59] Griffin M.D., Lutz W., Phan V.A., Bachman L.A., McKean D.J., Kumar R. (2001). Dendritic cell modulation by 1alpha,25 dihydroxyvitamin D3 and its analogs: A vitamin d receptor-dependent pathway that promotes a persistent state of immaturity *in vitro* and *in vivo*. Proc. Natl. Acad. Sci. USA.

[60] van Halteren A.G., van Etten E., de Jong E.C., Bouillon R., Roep B.O., Mathieu C. (2002). Redirection of human autoreactive T-cells upon interaction with dendritic cells modulated by tx527, an analog of 1,25 dihydroxyvitamin D(3). Diabetes.

[61] Berer A., Stockl J., Majdic O., Wagner T., Kollars M., Lechner K., Geissler K., Oehler L. (2000). 1,25-dihydroxyvitamin D(3) inhibits dendritic cell differentiation and maturation *in vitro*. Exp. Hematol..

[62] Pedersen A.W., Holmstrom K., Jensen S.S., Fuchs D., Rasmussen S., Kvistborg P., Claesson M.H., Zocca M.B. (2009). Phenotypic and functional markers for 1alpha,25-dihydroxyvitamin D(3)-modified regulatory dendritic cells. Clin. Exp. Immunol..

[63] Veldman C.M., Cantorna M.T., DeLuca H.F. (2000). Expression of 1,25-dihydroxyvitamin D(3) receptor in the immune system. Arch. Biochem. Biophys..

[64] Provvedini D.M., Tsoukas C.D., Deftos L.J., Manolagas S.C. (1983). 1,25 dihydroxyvitamin D3 receptors in human leukocytes. Science.

[65] von Essen M.R., Kongsbak M., Schjerling P., Olgaard K., Odum N., Geisler C. (2010). Vitamin D controls t cell antigen receptor signaling and activation of human t cells. Nat. Immunol..

[66] Kongsbak M., von Essen M.R., Levring T.B., Schjerling P., Woetmann A., Odum N., Bonefeld C.M., Geisler C. (2014). Vitamin D-binding protein controls t cell responses to vitamin D. BMC Immunol..

[67] Baeke F., Korf H., Overbergh L., van Etten E., Verstuyf A., Gysemans C., Mathieu C. (2010). Human t lymphocytes are direct targets of 1,25-dihydroxyvitamin D3 in the immune system. J. Steroid Biochem. Mol. Biol..

[68] Correale J., Ysrraelit M.C., Gaitan M.I. (2009). Immunomodulatory effects of vitamin d in multiple sclerosis. Brain.

[69] Bhalla A.K., Amento E.P., Serog B., Glimcher L.H. (1984). 1,25-dihydroxyvitamin D 3 inhibits antigen-induced t cell activation. J. Immunol..

[70] Lemire J.M., Adams J.S., Kermani-Arab V., Bakke A.C., Sakai R., Jordan S.C. (1985). 1,25-dihydroxyvitamin D 3 suppresses human t helper/inducer lymphocyte activity *in vitro*. J. Immunol..

[71] Kongsbak M., von Essen M.R., Boding L., Levring T.B., Schjerling P., Lauritsen J.P., Woetmann A., Odum N., Bonefeld C.M., Geisler C. (2014). Vitamin d up-regulates the vitamin D receptor by protecting it from proteasomal degradation in human cd4+ t cells. PLoS ONE.

[72] Rigby W.F.C., Stacy T., Fanger M.W. (1984). Inhibition of t lymphocyte mitogenesis by 1,25-dihydroxyvitamin D 3 (calcitriol). J. Clin. Invest..

[73] Rigby W.F., Denome S., Fanger M.W. (1987). Regulation of lymphokine production and human t lymphocyte activation by 1,25-dihydroxyvitamin D3. Specific inhibition at the level of messenger rna. J. Clin. Invest..

[74] Alroy I., Towers T.L., Freedman L.P. (1995). Transcriptional repression of the interleukin-2 gene by vitamin d3: Direct inhibition of nfatp/ap-1 complex formation by a nuclear hormone receptor. Mol. Cell. Biol..

[75] Cippitelli M., Santoni A. (1998). Vitamin D3: A transcriptional modulator of the interferon-gamma gene. Eur. J. Immunol..

[76] Rigby W.F., Waugh M., Graziano R.F. (1990). Regulation of human monocyte HLA-DR and CD4 antigen expression, and antigen presentation by 1,25-dihydroxyvitamin D3. Blood.

[77] Ikeda U., Wakita D., Ohkuri T., Chamoto K., Kitamura H., Iwakura Y., Nishimura T. (2010). 1alpha,25-dihydroxyvitamin D3 and all-trans retinoic acid synergistically inhibit the differentiation and expansion of th17 cells. Immunol. Lett..

[78] Joshi S., Pantalena L.C., Liu X.K., Gaffen S.L., Liu H., Rohowsky-Kochan C., Ichiyama K., Yoshimura A., Steinman L., Christakos S. (2011). 1,25-dihydroxyvitamin D(3) ameliorates th17 autoimmunity via transcriptional modulation of interleukin-17a. Mol. Cell Biol..

[79] Tang J., Zhou R., Luger D., Zhu W., Silver P.B., Grajewski R.S., Su S.B., Chan C.C., Adorini L., Caspi R.R. (2009). Calcitriol suppresses antiretinal autoimmunity through inhibitory effects on the th17 effector response. J. Immunol..

[80] Palmer M.T., Lee Y.K., Maynard C.L., Oliver J.R., Bikle D.D., Jetten A.M., Weaver C.T. (2011). Lineage-specific effects of 1,25-dihydroxyvitamin D(3) on the development of effector cd4 T cells. J. Biol. Chem..

[81] Boonstra A., Barrat F.J., Crain C., Heath V.L., Savelkoul H.F., O’Garra A. (2001). 1alpha,25-dihydroxyvitamin D3 has a direct effect on naive cd4(+) T cells to enhance the development of th2 cells. J. Immunol..

[82] Barrat F.J., Cua D.J., Boonstra A., Richards D.F., Crain C., Savelkoul H.F., de Waal-Malefyt R., Coffman R.L., Hawrylowicz C.M., O’Garra A. (2002). *In vitro* generation of interleukin 10-producing regulatory cd4(+) t cells is induced by immunosuppressive drugs and inhibited by T helper type 1 (th1)- and th2-inducing cytokines. J. Exp. Med..

[83] Pludowski P., Holick M.F., Pilz S., Wagner C.L., Hollis B.W., Grant W.B., Shoenfeld Y., Lerchbaum E., Llewellyn D.J., Kienreich K. (2013). Vitamin D effects on musculoskeletal health, immunity, autoimmunity, cardiovascular disease, cancer, fertility, pregnancy, dementia and mortality-a review of recent evidence. Autoimmun. Rev..

[84] Baeke F., Korf H., Overbergh L., Verstuyf A., Thorrez L., Van Lommel L., Waer M., Schuit F., Gysemans C., Mathieu C. (2011). The vitamin D analog, tx527, promotes a human cd4+cd25highcd127low regulatory t cell profile and induces a migratory signature specific for homing to sites of inflammation. J. Immunol..

[85] Vyas J.M., Van der Veen A.G., Ploegh H.L. (2008). The known unknowns of antigen processing and presentation. Nat. Rev. Immunol..

[86] Fabri M., Realegeno S.E., Jo E.K., Modlin R.L. (2011). Role of autophagy in the host response to microbial infection and potential for therapy. Curr. Opin. Immunol..

[87] Blanchet F.P., Piguet V. (2010). Immunoamphisomes in dendritic cells amplify tlr signaling and enhance exogenous antigen presentation on mhc-ii. Autophagy.

[88] Jagannath C., Lindsey D.R., Dhandayuthapani S., Xu Y., Hunter R.L., Eissa N.T. (2009). Autophagy enhances the efficacy of bcg vaccine by increasing peptide presentation in mouse dendritic cells. Nat. Med..

[89] Deretic V., Saitoh T., Akira S. (2013). Autophagy in infection, inflammation and immunity. Nat. Rev. Immunol..

[90] Cantorna M.T., Hullett D.A., Redaelli C., Brandt C.R., Humpal-Winter J., Sollinger H.W., Deluca H.F. (1998). 1,25-dihydroxyvitamin D3 prolongs graft survival without compromising host resistance to infection or bone mineral density. Transplantation.

[91] Hullett D.A., Cantorna M.T., Redaelli C., Humpal-Winter J., Hayes C.E., Sollinger H.W., Deluca H.F. (1998). Prolongation of allograft survival by 1,25-dihydroxyvitamin D3. Transplantation.

[92] Cantorna M.T. (2010). Mechanisms underlying the effect of vitamin D on the immune system. Proc. Nutr. Soc..

[93] Cantorna M.T., Mahon B.D. (2004). Mounting evidence for vitamin D as an environmental factor affecting autoimmune disease prevalence. Exp. Biol. Med. (Maywood.).

[94] Whitcomb J.P., Deagostino M., Ballentine M., Fu J., Tenniswood M., Welsh J., Cantorna M., McDowell M.A. (2012). The role of vitamin D and vitamin D receptor in immunity to leishmania major infection. J. Parasitol. Res..

[95] Ehrchen J., Helming L., Varga G., Pasche B., Loser K., Gunzer M., Sunderkotter C., Sorg C., Roth J., Lengeling A. (2007). Vitamin d receptor signaling contributes to susceptibility to infection with leishmania major. FASEB J..

[96] Bruce D., Whitcomb J.P., August A., McDowell M.A., Cantorna M.T. (2009). Elevated non-specific immunity and normal listeria clearance in young and old vitamin D receptor knockout mice. Int. Immunol..

[97] Yang H.F., Zhang Z.H., Chang Z.Q., Tang K.L., Lin D.Z., Xu J.Z. (2013). Vitamin D deficiency affects the immunity against mycobacterium tuberculosis infection in mice. Clin. Exp. Med..

[98] He Q., Ananaba G.A., Patrickson J., Pitts S., Yi Y., Yan F., Eko F.O., Lyn D., Black C.M., Igietseme J.U. (2013). Chlamydial infection in vitamin D receptor knockout mice is more intense and prolonged than in wild-type mice. J. Steroid. Biochem. Mol. Biol..

[99] Chen J., Waddell A., Lin Y.D., Cantorna M.T. (2014). Dysbiosis caused by vitamin D receptor deficiency confers colonization resistance to citrobacter rodentium through modulation of innate lymphoid cells. Mucosal. Immunol..

[100] Yuzefpolskiy Y., Baumann F.M., Penny L.A., Studzinski G.P., Kalia V., Sarkar S. (2014). Vitamin D receptor signals regulate effector and memory cd8 T cell responses to infections in mice. J. Nutr..

[101] Bruce D., Ooi J.H., Yu S., Cantorna M.T. (2010). Vitamin D and host resistance to infection? Putting the cart in front of the horse. Exp. Biol. Med. (Maywood).

[102] Nguyen N.L., Chen K., McAleer J., Kolls J.K. (2013). Vitamin D regulation of ox40 ligand in immune responses to aspergillus fumigatus. Infect. Immun..

[103] Enioutina E.Y., Bareyan D., Daynes R.A. (2009). Tlr-induced local metabolism of vitamin D3 plays an important role in the diversification of adaptive immune responses. J. Immunol..

[104] Enioutina E.Y., Bareyan D., Daynes R.A. (2007). Vitamin D3-mediated alterations to myeloid dendritic cell trafficking *in vivo* expand the scope of their antigen presenting properties. Vaccine.

[105] Kaposzta R., Tree P., Marodi L., Gordon S. (1998). Characteristics of invasive candidiasis in gamma interferon- and interleukin-4-deficient mice: Role of macrophages in host defense against candida albicans. Infect. Immun..

[106] Lucas R.M., Gorman S., Geldenhuys S., Hart P.H. (2014). Vitamin d and immunity. F1000Prime Rep..

[107] Zhao L.J., Zhou Y., Bu F., Travers-Gustafson D., Ye A., Xu X., Hamm L., Gorsage D.M., Fang X., Deng H.W. (2012). Factors predicting vitamin D response variation in non-hispanic white postmenopausal women. J. Clin. Endocrinol. Metab..

[108] Ahn J., Yu K., Stolzenberg-Solomon R., Simon K.C., McCullough M.L., Gallicchio L., Jacobs E.J., Ascherio A., Helzlsouer K., Jacobs K.B. (2010). Genome-wide association study of circulating vitamin D levels. Hum. Mol. Genet..

[109] Wang T.J., Zhang F., Richards J.B., Kestenbaum B., van Meurs J.B., Berry D., Kiel D.P., Streeten E.A., Ohlsson C., Koller D.L. (2010). Common genetic determinants of vitamin D insufficiency: A genome-wide association study. Lancet.

[110] Houghton L.A., Vieth R. (2006). The case against ergocalciferol (vitamin D2) as a vitamin supplement. Am. J. Clin. Nutr..

[111] Tai K., Need A.G., Horowitz M., Chapman I.M. (2008). Vitamin D, glucose, insulin, and insulin sensitivity. Nutrition.

[112] Bendik I., Friedel A., Roos F.F., Weber P., Eggersdorfer M. (2014). Vitamin D: A critical and essential micronutrient for human health. Front. Physiol..

[113] Holick M.F. (2004). Vitamin D: Importance in the prevention of cancers, type 1 diabetes, heart disease, and osteoporosis. Am. J. Clin. Nutr..

[114] Clemens T.L., Adams J.S., Henderson S.L., Holick M.F. (1982). Increased skin pigment reduces the capacity of skin to synthesise vitamin D3. Lancet.

[115] MacLaughlin J., Holick M.F. (1985). Aging decreases the capacity of human skin to produce vitamin D3. J. Clin. Invest..

[116] Vimaleswaran K.S., Cavadino A., Berry D.J., Whittaker J.C., Power C., Jarvelin M.R., Hypponen E. (2013). Genetic association analysis of vitamin D pathway with obesity traits. Int. J. Obes. (Lond.).

[117] Koutkia P., Lu Z., Chen T.C., Holick M.F. (2001). Treatment of vitamin D deficiency due to crohn's disease with tanning bed ultraviolet B radiation. Gastroenterology.

[118] Yamshchikov A.V., Desai N.S., Blumberg H.M., Ziegler T.R., Tangpricha V. (2009). Vitamin D for treatment and prevention of infectious diseases: A systematic review of randomized controlled trials. Endocr. Pract..

[119] Fetahu I.S., Hobaus J., Kallay E. (2014). Vitamin D and the epigenome. Front. Physiol..

[120] Carlberg C. (2014). Genome-wide (over)view on the actions of vitamin D. Front. Physiol..

[121] Carlberg C., Seuter S., de Mello V.D., Schwab U., Voutilainen S., Pulkki K., Nurmi T., Virtanen J., Tuomainen T.P., Uusitupa M. (2013). Primary vitamin D target genes allow a categorization of possible benefits of vitamin D(3) supplementation. PLoS ONE.

